# How Postmortem Redistribution of MDMA in Acute Alcohol-MDMA Combined-Use Rats Change under Effects of Alcohol

**DOI:** 10.1038/s41598-017-04416-y

**Published:** 2017-06-22

**Authors:** Man Liang, Jing Zhang, Na Zheng, Liang Liu

**Affiliations:** 1 Department of Forensic Medicine, Tongji Medical College, Huazhong University of Science and Technology, Wuhan, 430030 Hubei China; 2 Dongguan Public Security Bureau, Dongguan, 441900 Guangdong, China; 3 Department of Pathology, School of Basic Medical Sciences, Shen Zhen University, Shenzhen, 518066 Guangdong, China

## Abstract

MDMA is often taken recreationally with alcohol as combined-use. The objective was to determine MDMA postmortem redistribution (PMR) and corresponding effects in combined-style under different storage conditions. Steps were 20%-mixture of alcohol-water for initial 4 weeks to Group-A&B and intragastric infusions of MDMA (150 mg/kg) to Group-A later; in the same time, drinking pure water to Group-C&D first and then MDMA-fed to Group-C. The sacrificed rats were kept under different conditions for 10-d, during which the body fluids and tissues were collected on 15 continuous time-points and then detected. The MDMA concentrations were quite different along with postmortem interval (PMI) went by; the area under concentration-PMI curve significantly increased with combined-alcohol in comparison to MDMA alone, while that significantly decreased by lowering preservation temperature, allied with corresponding humidity. Combined-alcohol could exacerbate PMR of MDMA, as concentrations of combined-use rats’ samples were quite higher than mono-MDMA ones under any conditions, while different for body fluids and tissues; meanwhile lowering storage temperature could alleviate effects of alcohol. The study implies that in case of combined-use, the changes of concentrations are probably effected by some combined component, especially when come to identification of toxic level or even death.

## Introduction

3, 4-methylenedioxy-methamphetamine (MDMA), as an amphetamine derivative drug with entactogenic, empathogenic and hallucinogenic properties and one of the most popular amphetamine-type stimulants (ATSs), are often abused orally for recreational purposes, as consumed at rave parties in a combined abuse pattern, especially with cannabis, tobacco and ethanol; abuse of MDMA has increased throughout all over the world, and has become a global problem in recent years^1^. MDMA has a volume of distribution at steady state of 4.9 ± 2.6 L/kg, which belongs to substances having an apparent volume of distribution of more than 3–4 L/kg, and it is liable to postmortem drug redistribution^2^.

Recreational combined substances abuse is quite common in social parties and the trend spread during whatever age group, with users’ self-report euphoria, a sense of well-being and increased feelings of affiliation; while the regular combined-abuse over lifetime increased, most concerned with alcohol^3^. Among all, MDMA is frequently used in combination with others, and alcohol is one of the substances most frequently combined with it, for easily available and legal in most countries^4^. It seems that alcohol consumption enhances the risk of MDMA use; fatalities due to combined-use are occasionally encountered in toxicological practice^5^. Alcohol do exaggerate the effects of MDMA, while in rat models of acute MDMA-alcohol co-intoxication, alcohol may also potentiate PMR of MDMA. Recently, growing attention has been paid to combined drug use, especially since two or more substances use arouse a high-risk factor of substance-related problems.

Although in some studies, stimulants reduced the drunkenness scores, or the deleterious effects of alcohol on psychomotor performance^6^, significant pharmacological changes were not found in other investigations. However, data on this postmortem alcohol-concerned combination has seldom been investigated previously; how the alcohol would affect the PMR of MDMA when MDMA consumed in association with alcohol?

Drugs and alcohol often occur together in fatal poisonings, complicating the process of determining the cause of death; especially when found in concentrations generally regarded as toxic but not lethal, the question arises whether the combination of sub-lethal amounts and the drift of concentrations were the likely cause of death^7^. Although combined-use is very common, the understanding of the effects of substances-use, as well as the analysis of consequences of different drug-drug associations, deserved even more attention.

The purpose of this study was to investigate the changes of MDMA-concentration associated with co-consumption of psychoactive agent, alcohol, and to evaluate the different drifts of redistribution of MDMA in experimental rats during 10-day-storage and recognize the effects of alcohol on combined-interaction to MDMA alone. Besides, we investigated how PMR affected by alcohol and influenced by storage temperature and humidity when both conditions were changed.

## Materials and Methods

### Chemicals, Reagents and Solutions

MDMA as hydrochloride (Reference Standards, Lot No: 171242-201163) was obtained from Chinese Drugs & Biological Product Standardization Inst and the Internal Standard (I.S.); SKF_525A_ (propyl-adiphenine, Lot No: 028K1661) and trifluoroacetic acid (TFA, Lot No: 0001403330) were purchased from Sigma Co.; sodium chloride, sodium hydroxide, ethanol, methanol and I.S. isopropanol (all analytical grade) were purchased from Merck (Darmstadt, Germany). Water was purified by a Milli-Q water system (Millipore, Billerica, MA).

To prepare stock solutions, MDMA was dissolved in methanol at 2.0 mg/mL and SKF_525A_ at 1.0 mg/mL; and Isopropanol was prepared at 1.25 mol/L in deionized water. All the solutions were re-prepared every 2 weeks for stable quality controls.

### Instrumental conditions

A VARIAN CP-3800 gas chromatography (GC, Palo Alto, USA) equipped with a flame ionization detector (FID) and an electron capture detector (ECD) controlled by the Glaxie Work Station software was applied for quantitative measure of alcohol and MDMA.

Separation of alcohol (ethanol) was performed with a DM-WAX packed column (15 m × 0.25 mm × 0.25 μm). After 1 µL of sample injected into the column in splitless mode with an injector temperature at 200 °C, helium at a flow rate of 1.0 mL/min was used as carrier gas. The oven temperature was kept at 85 °C for 4 min, and then increased to 180 °C at 10 °C/min (held for 2 min); while the detector temperature was set at 220 °C. The flow rates of hydrogen, air and make-up gas (nitrogen) were adjusted to 30.0, 300.0 and 29.0 mL/min, respectively.

Determination of MDMA was performed by a CP8944 capillary column (30 m × 0.25 mm × 0.25 μm) with Helium at a flow rate of 1.0 mL/min as carrier gas. The oven temperature was set at 120 °C at first, then increased to 190 °C at 10 °C/min, and finally to 280 °C at 15 °C/min (held for 7 min) with the ECD set at 260 °C.

### Animals

All animal handling and experimental protocols were carried out in accordance with the Guide for the Care and Use of Laboratory Animals, and approved by the Research Council and Animal Care and Use Committee of Huazhong University of Science and Technology, Tongji Medical College, China (approval No. Y20100377). All efforts were made to minimize animal suffering and to reduce the number of animals used. 720 male SD rats, weighing 200 g ± 10 g, supplied by Experimental Animal Center, Tongji Medical College, Huazhong University of Science and Technology (China), were divided into four groups randomly (A, B, C and D, 180 rats per group, as described in Table 1). Group A and B rats were fed with 20% alcohol (ethanol-water) as the only drinking liquid for 4 weeks which were raised up as chronic active-drinking rats in these groups; meanwhile, the Group C and D were given pure water. Intragastric infusions of MDMA (150.0 mg/kg, approximately 3*LD_50_ according to preliminary experiment) were given to the chronic active-drinking rats and normal rats (Group A and C), respectively; and saline was offered to the other 2 groups (Group B and D) as blank. After these consecutive steps, acute combined drug intoxicated models (Group A), chronic active-drinking rats (Group B) and mono-MDMA intoxicated models (Group C) were set up, and Group D as control. 0.5 h after the intragastric infusion, all the rats were put to death by neck concussion and preserved at different conditions (Normal: 20 °C, humidity of 70%; Freeze: 0 °C, humidity of 50%) for 10 d. Alcohol (ethanol) in peripheral blood of Group A and B (n = 6) was determined immediately after death. In the 10-d period, samples of 4 groups, including heart blood, vitreous humor, urine, heart, liver, spleen, lung, kidney, brain, testicles and muscle, were collected at 15 continual time points (0 h, 2 h, 4 h, 10 h, 18 h, 24 h, 2 d, 3 d, 4 d, 5 d, 6 d, 7 d, 8 d, 9 d and 10 d, n = 6/time point•group). All the samples collected were kept at −70 °C until analysis.

**Table 1:** Grouping of Combined-Use Rats.

	MDMA (Experimental group)		Saline	
	20 °C, humidity of 70%	0 °C, humidity of 50%	20 °C, humidity of 70%	0 °C, humidity of 50%
20% alcohol-water (Experimental group)	A (180)		B (180)	
	90	90	90	90
Water	C (180)		D (180)	
	90	90	90	90

A: Acute Combined-use of Alcohol + MDMA.

B: Chronic Self-drinking of Alcohol.

C: Acute Use of MDMA.

D: Control.

### Sample preparation

Alcohol (ethanol) was extracted following these steps: 1 mL peripheral blood was placed in a 10-mL capacity glass tube fitted with a cap. After 15-min centrifugation at 2000 r/min, 200 µL of the upper layer serum was transferred into another glass tube, and 800 µL isopropanol-water solutions was added together. Then mixing for 10 s, aliquots of 1.0 μL were injected into the GC system.

Samples of body fluids (vitreous humor, heart blood and urine) and tissues (heart, liver, spleen, lung, kidney, brain, testicles and biceps femoris muscle) were extracted according to the following procedure: to 0.2 mL heart blood or urine, 50 μL vitreous humor or 1.0 g of each tissue, homogenized, and 20 μg SKF_525A_ (1.0 mg/mL*20 μL) were added to a 10-mL capacity centrifuge tube fitted with a cap, then added with NaOH (1.0 mol/L, adjusted precisely to pH10) and 1.0 g NaCl. Each sample, with 2 mL cyclohexane, was mixed and vibrated for 10 min on a vortex shaker; after centrifugation at 4800 r/min for 15 min, the organic layer was transferred into another tube and all the extraction steps were repeated once again. The organic layers evaporated dry by a Pressure Blowing Concentrator. 20 µL ethyl acetate and 20 µL TFA were added to the extractions; then the mixture, sealed, was derived by 60% microwave for 2 min and vaporized at 40 °C. The extractions were reconstituted by 20.0 μL methanol; aliquots of 1.0 μL were injected into the GC system.

### Statistical Analysis

Statistical evaluation of the data, which was performed according to SigmaStat 3.5 (SPSS Inc. 19.0) and software by two-way analysis of variance (ANOVA) and multivariate analysis of variance (MANOVA) at the 95% confidence interval, was used to determine whether MDMA levels of body fluids and tissues in 4 groups were statistically different during 10 d. Spearman correlation test was used to compare the individual sample levels of every group, in which P values less than 0.05 were considered to be statistically significant.

## Results

### Specificity

**Figure 1:**
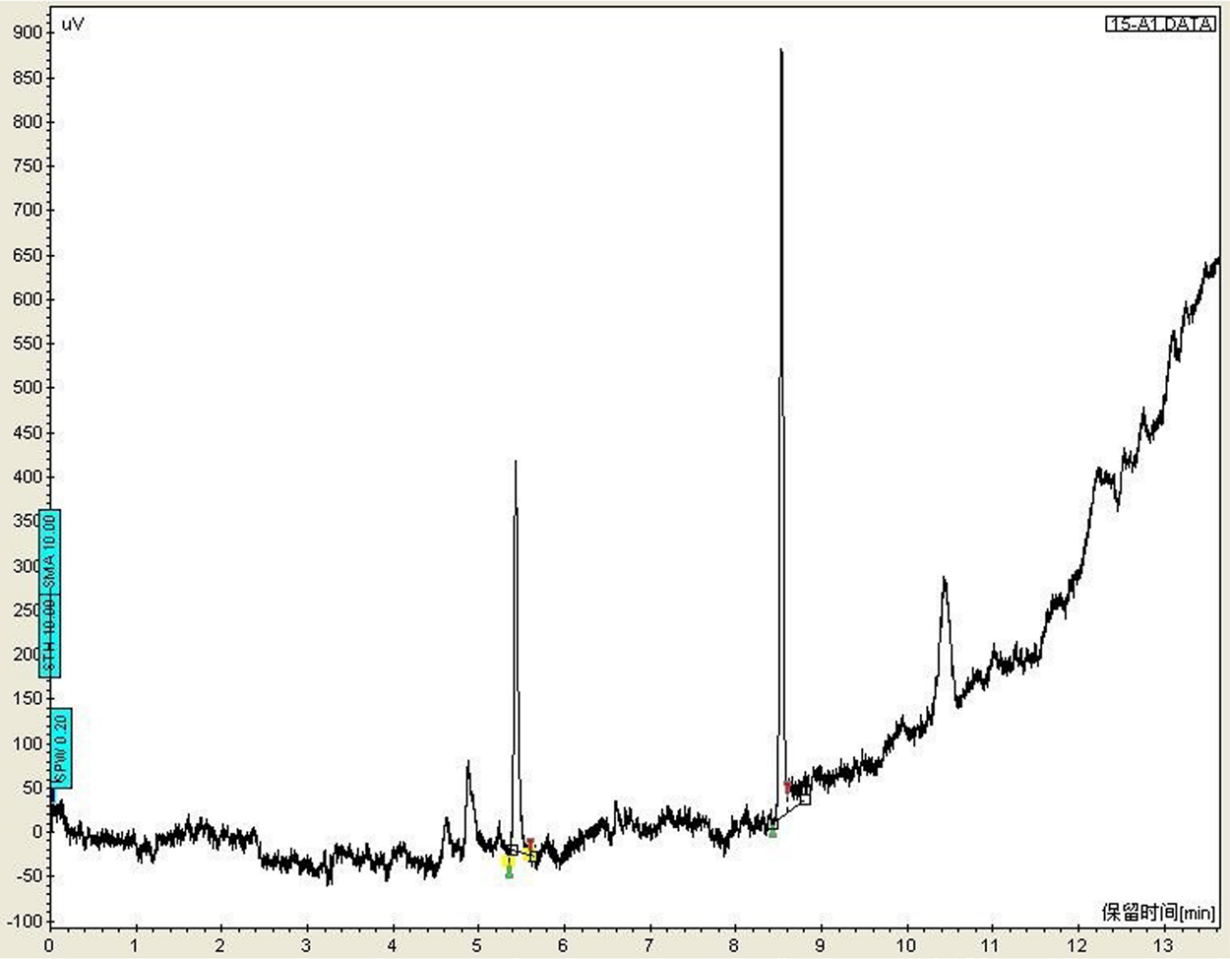
Chromatograms of ethanol and internal standard isopropanol from blood samples (T_R ethanol_: 5.496 min; T_R isopropanol_: 8.588 min).

**Figure 2:**
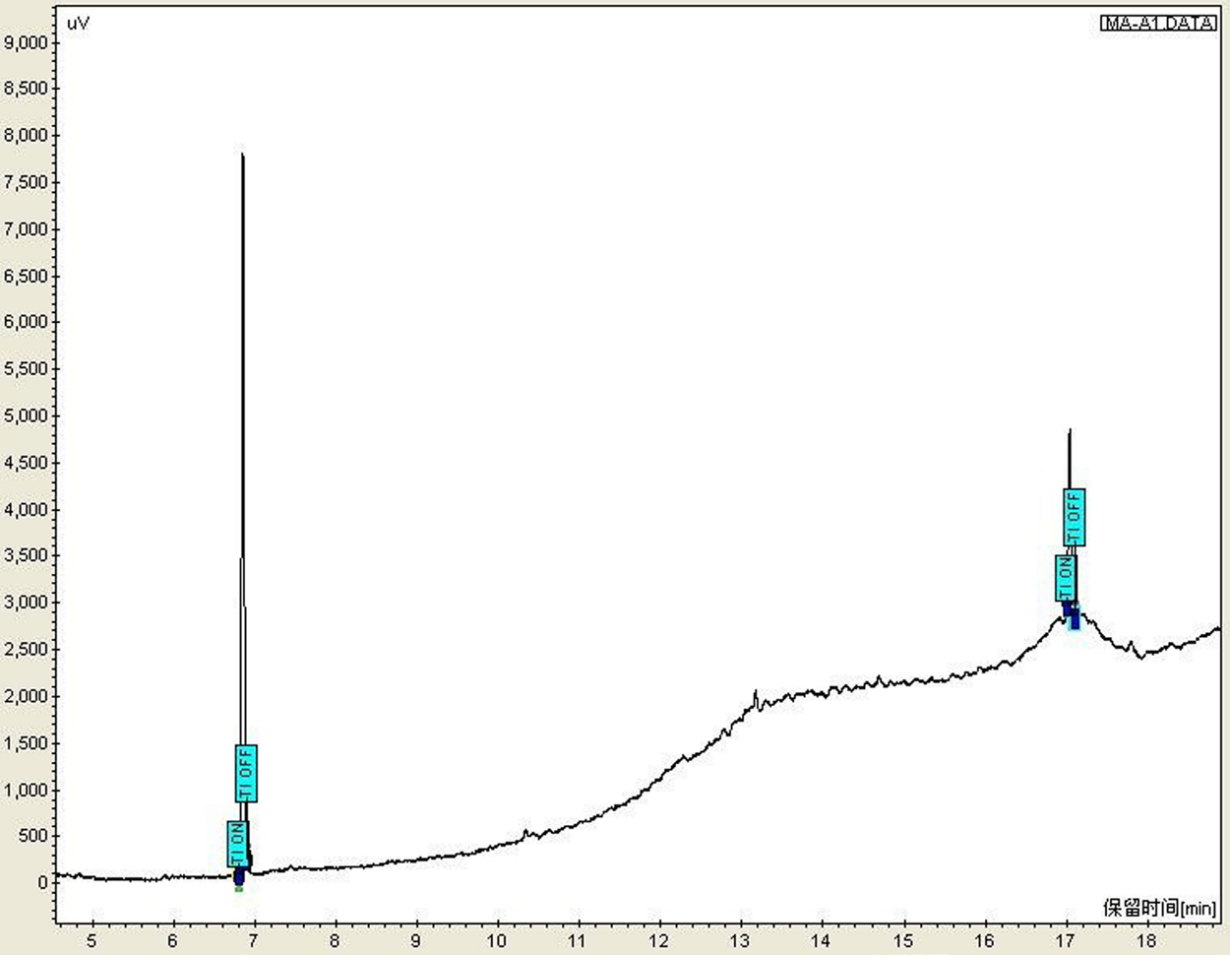
Chromatograms of extracts of MDMA and internal standard SKF_525A_ from blood samples (T_R MDMA_: 7.006 min; T_R SKF525A_: 17.016 min).

The chromatograms obtained from eight different blank plasma samples were studied to check the specificity. The chromatograms in Figs 1 and 2, showed exact separation of MDMA and alcohol from the internal standards and endogenous plasma substances was achieved; and at the retention times of MDMA and SKF_525A_, ethanol and isopropanol, no interfering peaks were observed respectively.

The mean BACs in Group A and B were around 11.385 ± 1.672 mg/100 mL and 10.852 ± 1.587 mg/100 mL. The significant difference of P > 0.05 by ANOVA which indicates there were no statistically significant differences of alcohol levels in peripheral blood of Groups A and B.

### Calibration and Limit of Detection of Ethanol (Alcohol)

By blank peripheral blood samples spiked with ethanol, alcohol calibration curve was prepared to cover the concentration range from 2.0, 5.0, 10.0, 20.0, 40.0 to 80.0 mg/mL with the I.S. isopropanol at the fixed concentration of 20.0 mg/mL. Calibration curve was set up by plotting drug concentrations against the peak-area ratio of ethanol and isopropanol, and each plotting was operated by the average of three runs at every concentration. No interfering peaks were detected during detection. Defined as a signal-to-noise ratio greater than 3, the limit of detection (LOD) was determined at 8 ng/mL for the preparation and detected condition. The linear regression slopes, intercepts and correlation coefficients of the calibration curves for alcohol were demonstrated in Table 2.

**Table 2:** Calibration equations and LOD of Alcohol in samples (n = 6).

ITEMS	DATA
Linear equation	Y_Blood_ = 0.0698 + 0.0142X
Concentration range	2.0~80.0 mg/mL
R	0.9963
LOD	8 ng/mL

Y stands for the peaks area ratio of alcohol and isopropanol; X stands for concentration of alcohol (mg/mL).

### Calibration and Limit of Detection of MDMA

Prepared with blank body fluids (heart blood, vitreous humor and urine) and tissues (heart, liver, spleen, lung, kidney, brain, testicles and biceps femoris muscle) spiked with MDMA, calibration curves were expressed to cover dynamic concentration ranges with the I.S. SKF_525A_ at the fixed concentration of 20.0 μg/mL or 20.0 μg/g. All the steps were repeated as aforementioned. The linear regression slopes, intercepts and correlation coefficients of the calibration curves from samples were presented correspondingly in Table 3.

**Table 3:** Calibration Equations and LOD of MDMA in Samples (n = 6).

Samples	Calibration equation	Calibration range	R	LOD
Vitreous humor	Y = 63.588X − 23.268	0.1~50.0 μg/mL	0.9992	5 ng/mL
Heart blood	Y = 50.784X − 7.7736	0.5~100.0 μg/mL	0.9953	8 ng/mL
Urine	Y = 48.474X + 2.5002	0.5~100.0 μg/mL	0.9971	5 ng/mL
Brain	Y = 45.523X + 1.2639	0.5~50.0 μg/g	0.9952	12 ng/g
Heart	Y = 53.823X − 0.4237	0.5~50.0 μg/g	0.9973	8 ng/g
Lung	Y = 43.644X − 2.5891	0.5~50.0 μg/g	0.9976	5 ng/g
Liver	Y = 68.351X − 1.1278	0.5~50.0 μg/g	0.9981	8 ng/g
Spleen	Y = 39.348X + 2.7963	0.5~50.0 μg/g	0.9939	5 ng/g
Kidney	Y = 35.731X − 1.5406	0.5~50.0 μg/g	0.9915	8 ng/g
Testicles	Y = 2.3146X + 0.6092	0.5~50.0 μg/g	0.9969	5 ng/g
Femoris muscle	Y = 3.4591X − 0.4629	0.5~50.0 μg/g	0.9992	5 ng/g

Y stands for the peaks area ratio of MDMA and SKF_525A_; X stands for concentration of MDMA (μg/mL and μg/g).

### Repeatability, Precision and Recovery

Added with MDMA at concentration of 0.5 μg/mL, 5.0 μg/mL and 20.0 μg/mL, blank peripheral blood samples were prepared for identification of repeatability and precision. They were obtained by plotting drug concentrations against the peak-area ratio of MDMA and SKF_525A_. By comparing spike area ratio at three different concentrations intra-day and inter-day, the repeatability of the method was estimated through precision calculation. The intra-assay precision was determined by spiked samples with respect to a calibration graph conducted in five days. The precision of the replicates was evaluated as the intra-day and inter-day coefficient of variation (%) according to relative standard deviation (RSD) shown in Table 4. The results indicate that the method presented good precision intra-day and inter-day.

**Table 4:** Repeatability, Precision and Recovery of MDMA (n = 5).

Samples	Standard concentration (µg/mL)	Precision intra-day		Precision inter-day%		Recovery	
		$\overline{x}\pm s$	RSD %	$\overline{x}\pm s$	RSD	$\overline{x}\pm s$	RSD%
Blood	0.5	0.517 ± 0.013	2.6	0.489 ± 0.025	5.1	103.6 ± 4.1	4.0
	10.0	10.461 ± 0.356	3.4	10.423 ± 0.386	3.7	102.4 ± 5.3	5.2
	50.0	51.935 ± 0.987	1.9	51.612 ± 1.18	2.3	105.3 ± 2.9	3.7

By adding standard stock solutions to blank plasma, the extraction recovery of MDMA and the I.S. in peripheral blood were analyzed at three levels of the concentration. The recovery of MDMA was calculated between 97.1% and 107.7% at different concentrations. Compared with other measures, the extraction steps responded to both the best recovery and chromatograms with less background noise for all the samples and fairly rapid and convenient prepared procedure. In forensic toxicology, alternative matrices to blood are useful in case of limited, unavailable or unusable blood sample, suspected postmortem redistribution or long drug intake-to-sampling interval.

### Postmortem Redistribution of MDMA

**Figure 3:**
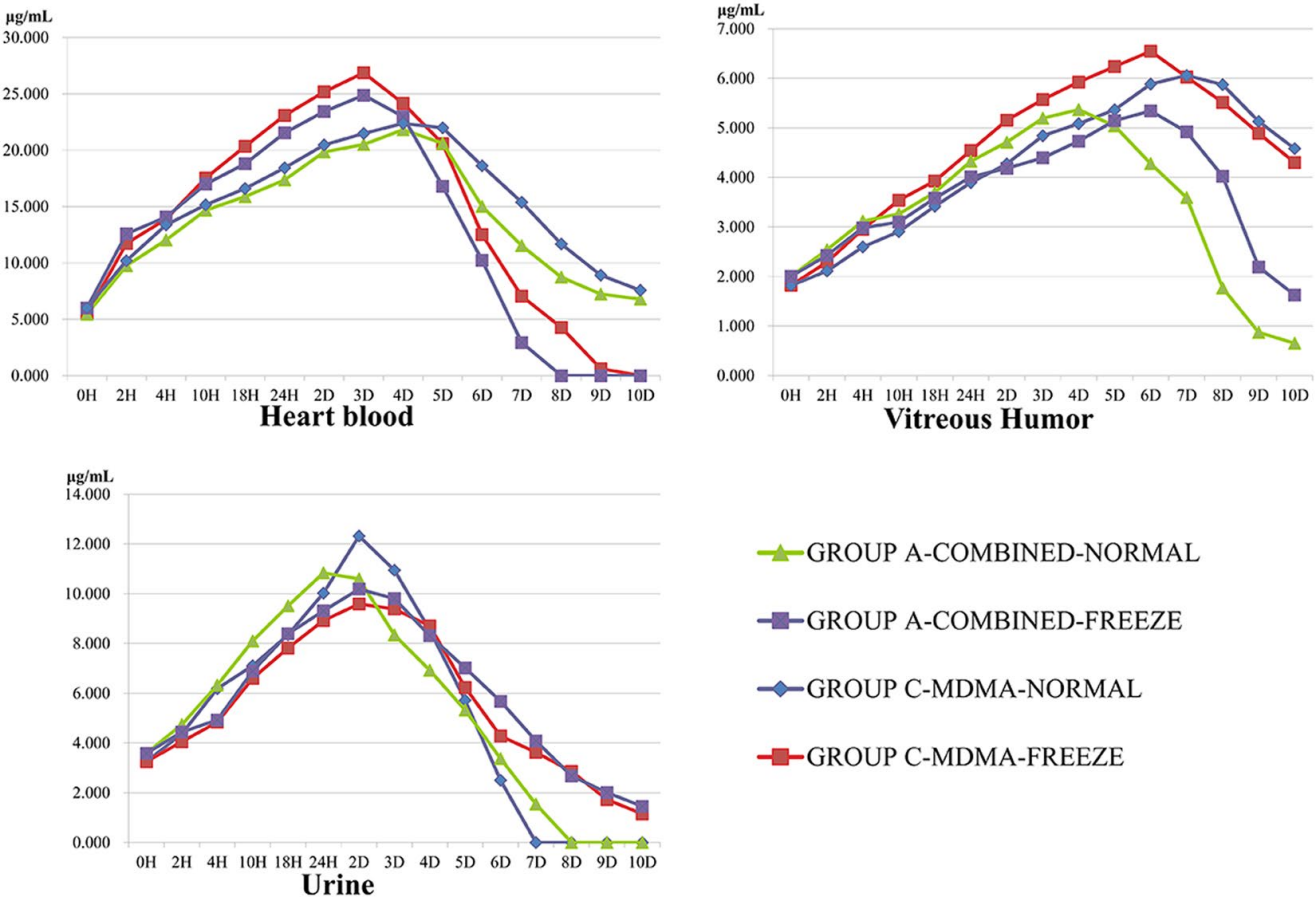
MDMA concentrations in body fluids in 10 d.

**Figure 4:**
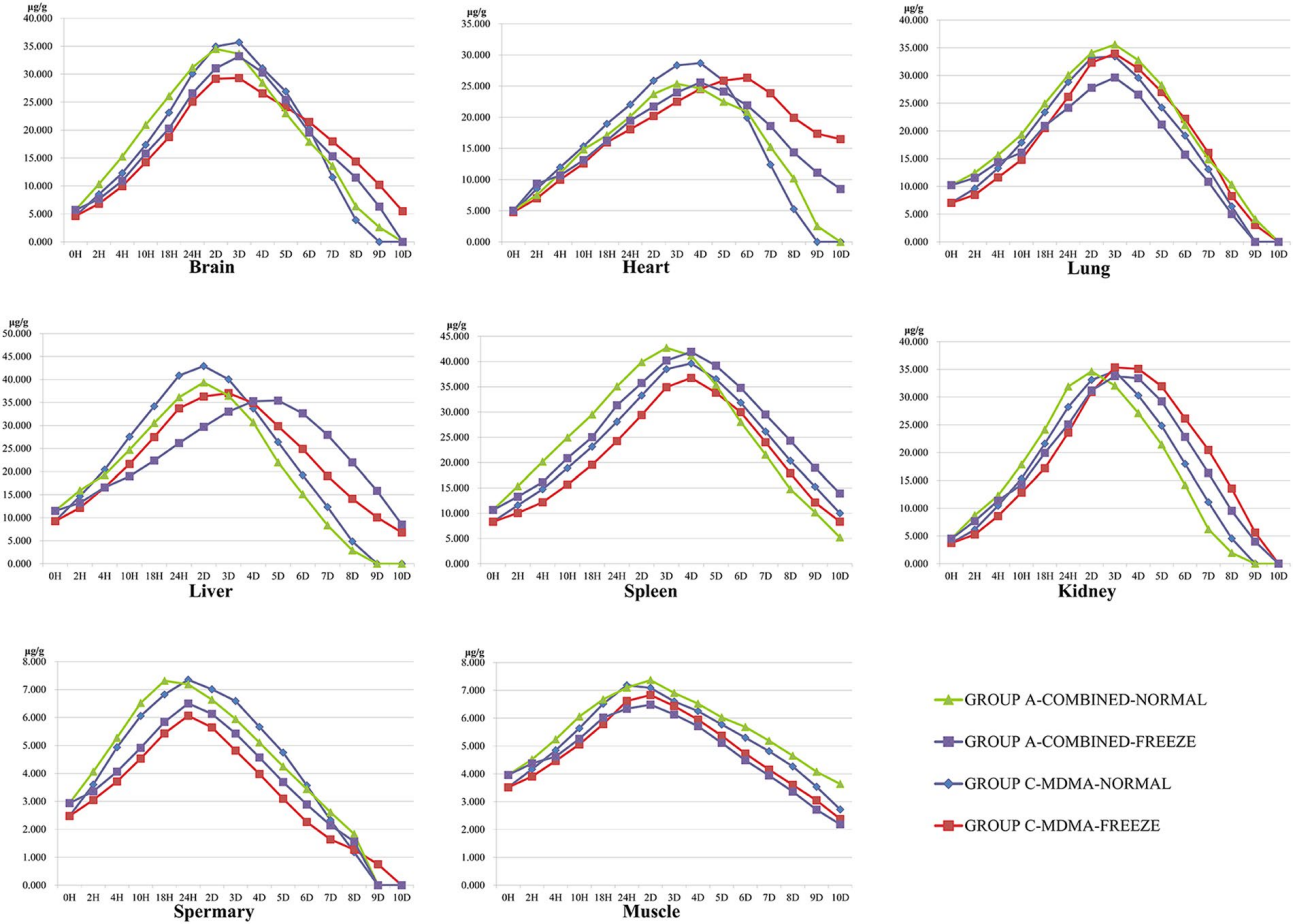
MDMA concentrations in tissues in 10 d.

The chronic self-drinking rats got more cognitive deficits than the rats fed on water-only. About 15 min after the intragastric infusion, Group A exhibited greater activity^8^, altered performance on the water feeding^9^, “hyper-attentiveness” to environment cues and an increase in breathing rate with obvious head wagging^10^. 5–15 min after intragastric administration, Group C responded similar symptoms to Group A. The concentrations of MDMA in samples of A and C groups’ rats are shown in Figs 3 and 4.

As shown in Figs 3 and 4, the MDMA concentration in samples varied dramatically in 10 d, and P < 0.01 by MANOVA indicates significant differences. Blood clotting in veins without injections of heparin into the bloodstream prior to death of all rats, clinical postmortem situations were reflected; and 200 μL heart blood was enough to conduct for determination of concentrations during 10-day postmortem interval. During the study sample analyses, a set of quality control (QC) samples were analyzed with each set of study samples and at least two-thirds of the QC samples had to be within ±15% in order for the sample batch to be accepted. Descriptive statistics (mean, standard deviation, and standard error of the mean) and exposure assessments (area under the curve or AUC) for the body fluids and tissue concentration data were calculated.

According to analysis of varied concentrations in samples, the changed trend during redistribution shared the same increase at the first stage and decrease in the latter period, even a few undetectable in the last days; the time point of concentration peaks in each sample were narrated in Table 5, and more samples displayed 1 or 2 days’ lag in freeze-storage group than in normal one when endured combined toxicity treat, except lung and femoral muscle, nevertheless, when treated with mono-MDMA, the amount of samples with lagging concentration peak were merely more than at-same-time ones (6/5).

**Table 5:** Comparison of Concentration Peaks between Groups during Redistribution.

Samples	Time Point of Concentration Peaks				Ratio of Concentration Peaks			
					Different Toxicated Style		Different Storage	
	Group A-Combined-Normal	Group A-Combined-Freeze	Group C-MDMA-Normal	Group C-MDMA-Freeze	Group A-Combined-Freeze/Group C-MDMA-Freeze	Group A-Combined-Normal/Group C-MDMA-Normal	Group A-Combined-Freeze/Group A-Combined-Normal	Group C-MDMA-Freeze/Group C-MDMA-Normal
Vitreous humor	4D	6D	6D	7D	0.881	0.820	0.995	0.926
Heart blood	3D	4D	3D	4D	1.025	0.926	0.899	0.812
Urine	24 H	2D	2D	3D	1.062	0.880	0.940	0.778
Brain	2D	3D	3D*	3D*	1.132	0.966	0.961	0.821
Heart	3D	4D	4D	6D	0.972	0.884	1.009	0.918
Lung	3D*	3D*	3D*	3D*	0.873^®^	1.064^®^	0.833^®^	1.015^®^
Liver	2D	5D	2D	3D	0.957	0.917	0.900	0.862
Spleen	3D	4D	4D*	4D*	1.141	1.078	0.981	0.928
Kidney	2D	3D	3D*	3D*	0.955^®^	1.000^®^	0.975^®^	1.020^®^
Testicles	18 H	24 H	24 H*	24 H*	1.073	0.995	0.889	0.825
Femoris muscle	2D*	2D*	24 H	2D	0.949^®^	1.025^®^	0.880^®^	0.951^®^

Setting ratios of concentration peaks in samples for example displayed in Table 4, the number of samples with lower concentration in mono-group was less than in combined-group at freeze storage condition (5/6), while even fewer at normal storage condition (4/7). No matter treated with combined style or mono-MDMA, majority samples in groups stored at normal condition were detected with higher concentrations than groups at freeze condition (10/1, 9/2).

Other than lung, kidney and femoris muscle, the ratios of samples’ concentrations in group A-combined-freeze to ones in group C-MDMA-freeze were always greater than in group A-combined-normal to group C-MDMA-normal for the rest specimen, meanwhile, the ratios of group A-combined-freeze to group A-combined-normal were also greater than group C-MDMA-freeze to group C-MDMA-normal though for different reasons.

## Discussions

In recent years, not only amphetamine-type stimulants (ATS), but also some other “club drugs”, both alone and in combination with alcohol, seemed to pose a much higher risk of poisoning, which was much more than expected from their constitution of a serious problem, reflected in the growing number of fatal poisonings^11^. It is noteworthy that even similar divisions result from both the median BAC analysis (Fig. 1) and the MDMA concentrations implied the combined-abuse may appear more toxic than mono-MDMA. Obviously, this division reflects not only the acute toxicity but also the manners of combined-use; some are deliberately taken in overdoses for intoxication purposes or with entertainment intent, which is evident from the varying proportions of purposes. Whatever the underlying mechanism, our results offer an alternative viewpoint on fatal drug-alcohol interactions with implications for drug effects.

### Redistribution by anatomical characteristics

Postmortem drug redistribution is a term used to describe drug concentration changes in the body following death, mainly thought to be due to passive diffusion. In this phenomenon, the concentration of drugs in the blood and other tissues can change up to 15-fold after death^12^. It is thought that all drugs will be affected to some extent by postmortem redistribution and the main contributing factors are (1) the time between death and sampling, (2) the site(s) of sampling (with femoral blood being the sample that is least affected by postmortem redistribution), (3) potential postmortem metabolism/production by either body enzymes or bacteria and finally, (4) physicochemical properties of the drug (such as pKa, LogP and most importantly volume of distribution (Vd))^2, 13^.

After administration to stomach, the diffusion pattern was observed depending on whether the MDMA solution was concentrated intra-gastrically or supra-diaphragmatically. The supra-diaphragmatic situation was about due to gastro-oesophageal reflux, which involved substantial diffusion into cardiac blood and muscle, lungs, liver and other organs. When the MDMA solution was concentrated more deeply in stomach, PMR did affect thoracic organs to a relative minor extent, and the intra-gastric solution diffused mainly into the closely adjacent organs, such as spleen. Along with the PMI, gastro-oesophageal reflux would happen even the MDMA solution deep enough as if increased pressure caused by decayed gases. There were also speculated that MDMA could easily diffuse into the thoracic and upper abdominal organs, and the amounts diffused increased erratically with the postmortem interval^14^.

While MDMA-alcohol co-treated, the MDMA concentrations in most tissues were substantial above the levels of mono-MDMA treated in early postmortem interval, but gradually decrease to sub-levels during the later postmortem interval. Generally, in both MDMA referring groups, MDMA concentrations were detected kept changing dramatically during the period, in both body fluids and tissues; in any postmortem interval, MDMA levels were higher in spleen, liver and kidney, yet relatively low in vitreous humor and biceps femoris muscle. These results in rats’ models suggested that the diffusion of MDMA out of stomach could lead to considerable postmortem redistribution and alcohol co-treat accelerates the diffusion. Thus, the redistribution and combined-use pattern should be taken into account in each specific sample in order to draw the corresponding conclusions when the concentration in a single sample is not sufficient for diagnosis and explanation.

### Specific redistribution change in samples

The relevance on the concentration changes in the sample and on the time of its storage period is an important factor which concerns obtaining relevant toxicological results and their interpretation. As for some specific samples, postmortem concentration increases and falls could have various explanations: (1) stability of the drug, (2) enzymatic and bacterial degradation or (3) diffusion.

### Heart

The importance of cardiac muscle levels in post-mortem toxicology has previously been investigated extensively, for cardiac stimulants^15^, and it seems cardiac muscle act a rather suitable substitution instead of heart blood for toxicological analysis when lack of blood with similar PMR concentrations at every time point as MDMA concentrations in cardiac is fairly stable due to pericardium.

### Brain

Though pKa of MDMA is 10.38, found totally in ionized form at physiological pH, and not been able to diffuse fluently to the brain^16^, a fact which suggests MDMA can easily pass through the blood-brain barrier (BBB) referring to the clinical effects, meanwhile MDMA, as lipophilic substances, is susceptible to postmortem redistribution, that active transport might take place as well^17^. Furthermore, the mechanism that MDMA possible affected the cerebral blood flow in a structure-dependent manner insure easily crosses to BBB. Besides, that alcohol can disrupt tight junctions in BBB and augment its permeability^18^ could induce the penetration of MDMA.

### Vitreous humor

In all the intoxicated rats, non-negligible MDMA levels were found in vitreous humor, though these levels were clearly lower than in the corresponding brain, which indicates that another mechanism other than pure diffusion from the brain should be assumed. One possible explanation is that there was direct or indirect reflux into the naso-pharynx with diffusion of MDMA into the sinuses, the skull base and the orbitae^5^.

Even combined with alcohol under less humidity, the (relatively minor) MDMA increases in vitreous humor levels cannot be due exclusively to dehydration, though the rise in creatinine concentration in vitreous humor at increasing postmortem interval due to dehydration has been confirmed^2^. As for the decrease in the latter period, it may be attributed predominantly to spontaneous chemical hydrolysis.

### Biceps femoris muscle

In all the experiments performed, we observed that the MDMA concentrations in the biceps femoris muscle, without adjacent organs, were the least subject to postmortem diffusion, and thus remained relatively stable after death^19, 20^. Therefore, biceps femoris muscle can be an interesting specimen when the usual samples for lacking drug assay and unknown postmortem interval or when advanced putrefaction has occurred. This has previously been suggested for methamphetamine and amphetamine^21^.

### Kidney

For the kidney tissue extensively permeated by urine, the MDMA concentrations in the kidney were the highest of all samples which levels can be interpreted as due to “inherent contamination” and enrichment^7^.

### Testicles

The MDMA levels in testicles were very low and often near or just below the quantification limit. Besides blood-testis barrier, we cannot exclude the possibility that sampling 0.5 h after administration provides insufficient time for MDMA absorption and gain access into this certain tissue.

In all, as the ratios of the MDMA concentration levels in tissue to blood were gradually higher, and then lower for most organs, accumulation in tissues were variated. Referring to the positive correlation between the MDMA levels in postmortem blood via cardiac muscle, lungs and liver, we can assume stronger postmortem diffusion between these organs with richer blood supply and cardiac blood stagnant; and the significant increases in postmortem MDMA liver concentrations can partially be explained as a result of diffusion from the residue in stomach.

### Interaction by combination

An overall interpretation to account for interactions between alcohol and MDMA was related to the absorption, distribution, redistribution and elimination of MDMA^22^.

There are a more important pharmacodynamic effects for the interaction between alcohol and drug in fatal poisonings than pharmacokinetic^7, 15^; an increase of bioavailability has been proposed by alcohol, for a mechanism partly attributed to the acceleration of chemical hydrolysis being reflected to magnitude changes in initial MDMA distribution, was consistent with findings of a previous study in which alcohol and methamphetamine were co-administered^23, 24^.

CYP enzymes in hepatocytes are responsible for MDMA metabolism and contribute in part to alcohol metabolism, hence alcohol might inhibit the antemortem metabolism of MDMA by competitive inhibition of cytochrome P450^25^. Therefore, it seems likely that alcohol has facilitated the initial lowering of MDMA at 0 h and the MDMA concentrations in Group A were higher than Group C in all samples indeed. Besides, alcohol was previously shown to induce a fluidizing effect on the intestinal membrane, and thereby to facilitate the absorption of other molecules^26^, hence it is suspicious whether this is plausible with MDMA.

Alcohol has been suggested that ‘potentiates the fatal toxicity’ of certain substances during interaction, as measured by the ratio of the mean drug concentration in fatal drug poisonings with and without alcohol^27^. In earlier interaction studies on postmortem data, the outcome measure has been drug concentration in the presence and absence of alcohol, rather than vice versa^2^. Additionally, the alcohol presence in biological specimens is a potential factor affecting analyses stability and its concentration, respectively^28^. The finding, that as ratios of concentration peaks in samples displayed in Table 5, none of ratios of the combined-group via mono-group samples’ concentration equaled to 1, which specifically, the number of samples with lower concentration in mono-group was less than in combined-group at freeze storage condition (5/6), while even fewer at normal storage condition (4/7), has no contradiction with result of mono and combined having similar redistribution pattern but different concentrations. Based on the ratios of almost half samples’ concentrations in group A-combined-freeze to group C-MDMA-freeze greater than 1, it seems that alcohol is able to slightly increase the levels of MDMA in heart blood, urine, brain, spleen and testicles, yet MDMA was reduced by combined-alcohol in vitreous humor, heart, lung, liver, kidney and femoris muscle with the uncertain mechanism; while at normal storage with alcohol, the ratio of the amounts of samples for the MDMA reduced via increased was 7/4.

### Effects of storage conditions-temperature

The storage conditions (temperature and corresponding humidity) are the crucial factor concerning the quantitative drug presence in the sample, as a circumstance determining the samples content stability and preserving of initial and actual result of testing^29^.

As mentioned above, diffusion is thought to be the most likely explanation in postmortem changes^13^. It is likely that in 10 days after euthanasia the equilibrium between blood and tissue concentrations may not have been reached and the kinetics kept varying 0.5 h after injection, which seemed to indicate that the diffusion and elimination phase was ongoing. It is also possible that this equilibrium between blood and tissues was modified after death due to the ongoing storage temperature in the postmortem environment for possible movement of drugs.

Different postmortem environment would modify the current theories on how drugs in general redistribute by influence of combination of alcohol. In our study, they are (1) physically, the motive force for the postmortem equilibrium of the drug in tissue compartments changes by the temperature, as the lower the storage temperature is, the more the force reduces; (2) passive diffusion of the drug down a concentration gradient (either from neighboring tissue, from the gastrointestinal tract or through blood vessels). As for different tissues with different water content, when stored at 0 °C, water froze to ice, density of which is lower than water, which led to varied changes of mean density of different tissues. These changes would affect the equilibrium and passive diffusion; on the base of the changes, if there is postmortem movement, it would be expected to move less from areas of high concentration to areas of low concentration at 0 °C. Thus, the final redistribution findings, as shown in Figs 3, 4 and Table 5, were complex under these conditions since the presence of ethanol exhibited varied effects on stability in different samples and freeze storage affected the remaining amount of MDMA in all kinds of samples.

Another possible explanation of the effect observed is the change of the stability of the sample which represents a colloidal system^30^. The samples displayed a significant increase, and then decrease for the different time period which can affect the interpretation of the results. The presence of ethanol additionally increases the risk of concentration changes of MDMA in some cases, with lower stability observed (Table 5). The combination of ethanol and normal temperature destabilizes the samples and increases the risk of changing remaining MDMA concentration in the sample (estimated by the ratios of concentration peaks in samples), which is correlated to ethanol concentration.

Besides to some users’ self-report euphoria and an increased perception and feeling of closeness to others, MDMA users may develop acute complications with potential fatal consequences when taken in warm environments^12^. As for corpse, the lower storage temperature stopped postmortem redistribution of MDMA partly at 0 °C and humidity of 50%, showing less change of concentrations than 20 °C and humidity of 70%, no matter in any sample. In our study, other than lung, kidney and femoris muscle, the ratios of samples’ concentrations in group A-combined-freeze to ones in group C-MDMA-freeze were always greater than in group A-combined-normal to group C-MDMA-normal for the rest specimen. The results implied that the combination effect played an even stronger role in MDMA redistributions than storage temperature.

## Conclusion

The median BAC was significantly higher in case with combined-use than in those with alcohol alone and the same with the concentration of MDMA than mono-MDMA, suggesting a positive concentration-effect relationship. The results reflect not only the acute toxicity of a given drug-alcohol combination but also the manners of use and abuse of these drugs, while the cause of death was concluded due to the interaction of MDMA and ethanol. So, the effects of putrefactive changes in PMR should be considered during further toxicological evaluation.
